# Decreased tissue oxygenation in newborns with congenital heart defects: a case-control study

**DOI:** 10.3325/cmj.2018.59.71

**Published:** 2018-04

**Authors:** Petja Fister, Domen Robek, Darja Paro-Panjan, Uroš Mazić, Helena Lenasi

**Affiliations:** 1 University Medical Centre Ljubljana, Division of Pediatrics, Department of Neonatology, Ljubljana, Slovenia; 2 University Medical Centre Ljubljana, Division of Pediatrics, Department of Cardiology, Ljubljana, Slovenia; 3 Institute of Physiology, Medical Faculty, University of Ljubljana, Ljubljana, Slovenia

## Abstract

**Aim:**

To compare regional tissue oxygenation (rSO_2_) in the brain, intestine, and kidney between newborns with and without congenital heart defects (CHD).

**Methods:**

This observational case-control study was conducted at the Neonatal Deparetment of Children's Hospital Ljubljana between December 2012 and April 2014. It included 35 newborns with CHD and 30 healthy age- and sex-matched controls. CHD were assessed echocardiographically and divided into acyanotic and cyanotic group. RSO_2_ in the brain, intestine, and kidney was measured using near-infrared spectroscopy (NIRS). Simultaneously, heart rate (HR), breathing frequency (BF), mean arterial blood pressure (MAP), and arterial oxygen saturation (Sao_2_) were recorded.

**Results:**

Newborns with CHD had significantly lower rSO_2_ in the left brain hemisphere (67 ± 11% vs 76 ± 8%, *P* = 0.004), right brain hemisphere (68 ± 11% vs 77 ± 8%, *P* < 0.001), and the kidney (68 ± 13% vs 77 ± 10%, *P* = 0.015). RSO_2_ in the intestine did not significantly differ between the groups. HR, MAP, and Sao_2_ also did not differ between the groups, whereas BF was significantly higher in the CHD group (57 ± 12 vs 39 ± 10 breaths/min, *P* < 0.001). Between cyanotic and acyanotic group, we found no significant differences in rSO_2_ of any tissue.

**Conclusions:**

Monitoring tissue oxygenation by NIRS could enable a timely detection of hemodynamically important CHD.

Congenital heart defects (CHD) occur in about 1% of newborns, causing blood flow obstruction or blood shunting of different degree (1,2). Right-to-left shunts increase end-diastolic pressure of the left ventricle and are associated with cyanosis. Left-to-right shunts, on the other hand, increase right ventricle end-diastolic pressure and lung perfusion (1,2). Critical CHD can cause diastolic dysfunction, leading to heart failure, organ hypoperfusion, and oxygen-deficiency in target tissues, despite their normal arterial oxygen saturation (1,2).

Currently no clinical tool can non-invasively assess appropriate tissue oxygen supply in real-time. Namely, patients in a compensated condition may have compromised tissue oxygenation but still exhibit normal hemodynamic variables. A promising method for detecting subtle alterations in regional tissue oxygenation (rSO_2_) is near-infrared spectroscopy (NIRS). The method assesses oxygenated and deoxygenated hemoglobin by comparing scattered and absorbed near-infrared light (3-7). Rather than assessing cerebral perfusion, NIRS estimates tissue oxygenation that reflects the fine tuning between the microcirculatory blood flow and oxygen supply to the tissue and oxygen consumption by the tissue (3,5-7).

Although many studies have used NIRS to investigate brain oxygen supply in newborns with CHD (8,9), none of them have compared it between patients and healthy controls. Also, most of the studies estimated the outcome of different therapeutic procedures in regard to rSO_2_ (8-14), but did not evaluate the preoperative brain condition in newborns with CHD, although these newborns have been shown to have delayed brain development (1). Inadequate perfusion may more seriously affect organs other than the brain, such as the intestine and kidney, since the brain is subjected to powerful autoregulation mechanism. Yet, there are limited data on the regional oxygenation of organs in newborns with CHD (4,9,12,15-17). Also, no study separately assessed tissue oxygenation in acyanotic and cyanotic CHD.

We hypothesized that rSO_2_ would be lower in newborns with CHD compared to healthy newborns. We also hypothesized that it would be lower in cyanotic than in acyanotic group due to mixing of unsaturated blood with systemic circulation. The aim of our study was to noninvasively assess the brain, kidney, and intestine oxygenation in newborns with critical structural CHD and in healthy newborns without CHD using NIRS.

## Patients and methods

### Patients

This observational retrospective case-control study (level of evidence 2B) was conducted at the Neonatal Department of Children’s Hospital Ljubljana, Slovenia, from December 2012 to April 2014. The newborns' parents signed the informed consent, and the ethical approved was obtained from the National Ethics Committee (August 16, 2011; approval No. 123/08/11). The investigation conforms to the Declaration of Helsinki principles.

We included 35 term newborns with critical structural CHD of different types who required surgical or transcathetral procedure in the first year of life. CHD were diagnosed by pediatric cardiologists using clinical examination and echocardiography. The exclusion criteria were pulmonary or neurological disease, perinatal asphyxia, acute illness, prematurity, and congenital abnormality other than CHD.

We divided the CHD group into two subgroups: the group where the clinical signs are accompanied by cyanosis (16 patients), including the following defect types: tetralogy of Fallot (n = 4), pulmonary atresia (n = 3), double outlet of the right ventricle (n = 3), transposition of the great arteries (n = 3), atrioventricular septal defect (n = 3); and the group without cyanosis (19 patients), including the following defect types: ventricular septal defect (n = 7), pulmonary stenosis (n = 5), aortic stenosis or coarctation of the aorta (n = 5), hemodynamically important atrial septal defect (n = 2).

The control group comprised 30 healthy age- and sex-matched newborns without CHD hospitalized at our department. Their venous blood was taken to detect conditions not affecting the investigated outcome, such as infection, benign systolic murmur, poor feeding or weight gain, or congenital urinary tract defects. The exclusion criteria were the same as for the study group.

### Methods

CHD were diagnosed by ALOKA ProSound Alpha 10 ultrasound system (Tokyo, Japan) by a pediatric cardiologist specialized for ultrasonographic examinations. Tests were performed with newborns lying comfortably in a peaceful environment. Electrodes (IntelliVue MP 50, Philips, Boeblingen, Germany) were placed on the thoracic wall to measure the heart rate (HR), breathing frequency (BF), mean arterial blood pressure (MAP), and hemoglobin oxygen saturation in arterial blood (Sao_2_) assessed by noninvasive pulse oximetry.

Venous blood samples were analyzed in the laboratory of the Department of Clinical Biochemistry, University Children's Hospital, University Medical Centre Ljubljana. Serum glucose concentration was measured by an enzymatic UV test (hexokinase method) using AU 400 analyzers (Beckman Couter Inc., Maryfort, Ireland); hemoglobin and hematocrit using Sysmex XT 2000i analyzer (Sysmex Corporation, Kobe, Japan); and partial pressure of CO_2_ using Cobas b 221 blood gas analyzer (Roche Diagnostics GmbH, Mannheim, Germany).

RSO_2_ was measured using *in vivo* optical spectroscopy (INVOS Cerebral/Somatic oximeter, 5100C Monitor, Somanetics, Minneapolis, MN, USA). The system consists of four sensors that are fastened to the skin. Each sensor has a near-infrared light-emitting diode and two detectors of the light reflected from the superficial and deeper tissues. These two signals are subtracted to assess deep tissue oxygenation.

The neonatal NIRS probes were placed on the skin and attached with elastic bandage as follows: for brain rSO_2_ assessment, symmetrically over the left and right frontal head area; for intestinal rSO_2_ assessment, to the abdomen 3 cm to the left of the umbilicus; for kidney rSO_2_ assessment, lumbally 2 cm from the medial line. RSO_2_ was calculated from the differential signals obtained from these two sensors, expressed as the venous-weighted per cent oxygenated hemoglobin [oxygenated hemoglobin/total hemoglobin (oxygenated hemoglobin + deoxygenated hemoglobin)] (3,4,18). To calm the newborn down and record a stable signal, the NIRS recording was left running for 30 minutes after the probes had been attached; subsequently a five-minute sample was exported for further analysis. All other vital functions measurements were recorded at the midpoint of the five-minute recording and marked in the patient’s documentation. RSO_2_ was measured simultaneously at all acquisition sites at the preset device sampling rate of approximately six seconds. Acquired values from each channel were averaged over five minutes, using licensed version of INOVOS Analytics tool. Data were stored on personal computer for further analysis.

### Statistical analysis

The normality of distribution was tested by Shapiro-Wilk test, using a significance level of 0.05. Descriptive statistics were reported and the numerical variables were summarized as means ± standard deviations (SD) or medians and ranges, while categorical variables were summarized as proportions (with their 95% CI). Significance of the relationship between normally distributed numerical variables was tested by Welsh two-sample *t* test; between not normally distributed variables with Mann-Whitney test; and between categorical variables with χ^2^ test with Yates’ continuity correction. To control for false positives, the *P* values were adjusted using a multivariate permutation procedure (19). The correlation between numerical variables was evaluated using Spearman’s rank correlation analysis. Adjusted *P* lower than 0.05 was considered significant. Statistical analysis was performed using R statistical software, version 3.0.3.

## Results

There were 23 (66%) boys in the CHD group and 16 (53%) in the control group. CHD patients and controls did not significantly differ in demographical characteristics (Table 1) or in laboratory and physiological parameters; only BF was significantly higher in newborns with CHD (57 ± 12 vs 39 ± 10 breaths per minute, *P* < 0.001) (Table 2).

**Table 1:** Clinical characteristics of the newborns with congenital heart defect (CHD) and controls (mean ± standard deviation [SD] or median and range)

Characteristic	CHD (n = 35)	Controls (n = 30)	Difference (controls-CHD) (95% confidence intervals)	Adjusted *P* (Welsh two-sample *t* test or Mann-Whitney test*)
Gestational age in weeks, median (range)	38 (37-39)	38 (37-39)	0.3 (-0.7-1.3)	>0.99*
Birth weight in grams, mean ± SD	3157 ± 670	3197 ± 563	40 (-266-346)	>0.99
Head circumference in centimeters, median (range)	34 (34-35)	34 (34-35)	-0.06 (-0.96-0.85)	>0.99*
Apgar score 5 min, median (range)	9 (8-9)	9 (9-10)	0.41 (-0.21-1.03)	>0.99*
Age in days, median (range)	15 (10-20)	11 (8-14)	-3.77 (-9.35-1.82)	>0.99*

**Table 2:** Laboratory and physiological parameters in newborns with congenital heart defect (CHD) and controls (mean ± standard deviation [SD] or median and range)

Parameter	CHD (n = 35)	Controls (n = 30)	Difference (controls-CHD) (95% confidence intervals)	Adjusted *P* (Welsh two-sample *t* test or Mann-Whitney test*)
	mean ± SD	mean ± SD/ median (range)		
Serum glucose (mmol/L)	4.3 ± 0.8	4.4 ± 1.15	0.1 (-0.6-0.8)	>0.99
Hemoglobin (g/L)	157 ± 32	168 ± 39	11.0 (-7.0-29.0)	>0.99
Hematocrit (%)	0.46 ± 0.1	0.46 ± 0.1	0.0 (-0.05-0.05)	>0.99
Carbon dioxide partial pressure (kPa)	5.4 ± 1.1	5.2 ± 0.6	-0.1 (-0.8-0.5)	>0.99
Heart rate (beats/min)	140 ± 19	136 ± 19	-4.44 (-14-5)	>0.99
Breathing frequency (breaths/min)	57 ± 12	39 ± 10	-17.68 (-23.31 to -12.06)	<0.001
Arterial blood oxygen saturation (%), median (range)	95 (93-96)	96 (94-98)	1.11 (-1.31-3.54)	>0.99*
Mean arterial pressure (mmHg)	57 ± 10	56 ± 7	-1.24 (-6.98-4.49)	>0.99

**Figure 1:**
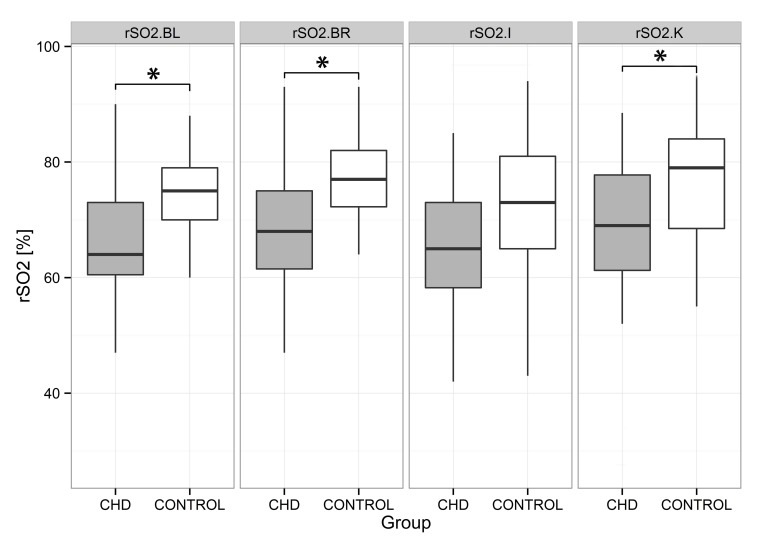
Regional tissue oxygenation (rSO_2_, %) in the left (rSO_2_.BL) and right brain hemisphere (rSO_2_.BR), intestine (rSO_2_.I), and kidney (rSO_2_.K) in newborns with congenital heart defect (CHD, gray bars) and healthy newborns (control, white bars). Mean values and standard deviation are presented. *P ≤ 0.01 (Welsh two-sample *t* test).

Newborns with CHD had significantly lower rSO_2_ in the left (67 ± 11% vs 76 ± 8%) and right (68 ± 11% vs 77 ± 8%) brain hemisphere than controls (delta 8.6 and 9.0; 95% CI 3.9 to 13.4 and 4.4 to 13.7; adjusted *P* = 0.004 and *P* < 0.001, respectively, Figure 1). They also had significantly lower rSO_2_ in the kidney (68 ± 13% vs 77 ± 10%; delta 9.0; 95% CI 3.2 to 14.8; adjusted *P* = 0.015) and the intestine, but the latter difference was not significant (65 ± 13% vs 72 ± 15%; delta 7.1; 95% CI 0.1 to 14.1; adjusted *P* = 0.174) (Figure 1).

**Figure 2:**
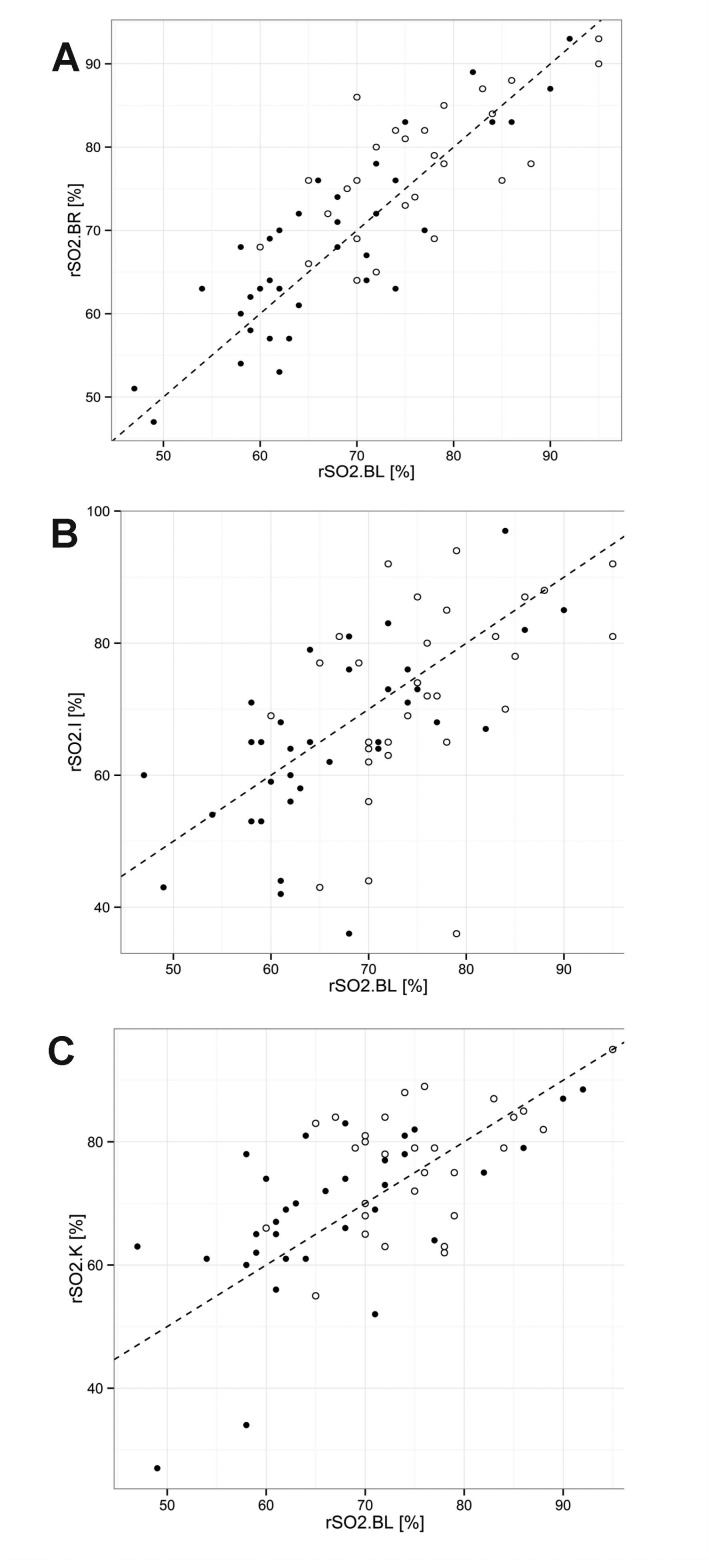
Correlation between (**A**) the regional tissue oxygenation (rSO_2_) in the left (rSO_2_.BL) and the right brain hemisphere (rSO_2_.BR), (**B**) rSO_2_.BL and the intestine rSO_2_ (rSO_2_.I) and (**C**) rSO_2_.BL and kidney rSO_2_ (rSO_2_.K) in newborns with congenital heart defect (CHD, closed circles) and controls (open circles). Spearman’s correlation coefficients were (**A**) r = 0.80 (*P* < 0.001), (**B**) r = 0.65 (*P* < 0.001), and (**C**) r = 0.70 (*P* < 0.001).

The rSO_2_s in the left and right brain hemisphere (r = 0.80; *P* < 0.001), intestine (r = 0.65; *P* < 0.001), and kidney (r = 0.70; *P* < 0.001) were significantly positively correlated (Figure 2). The strongest correlation was observed between the left and the right brain hemisphere (Figure 2A). The main differences in rSO_2_ measurements were centered around 0, while the lowest variability was observed for the differences between the left and the right brain hemisphere.

**Figure 3:**
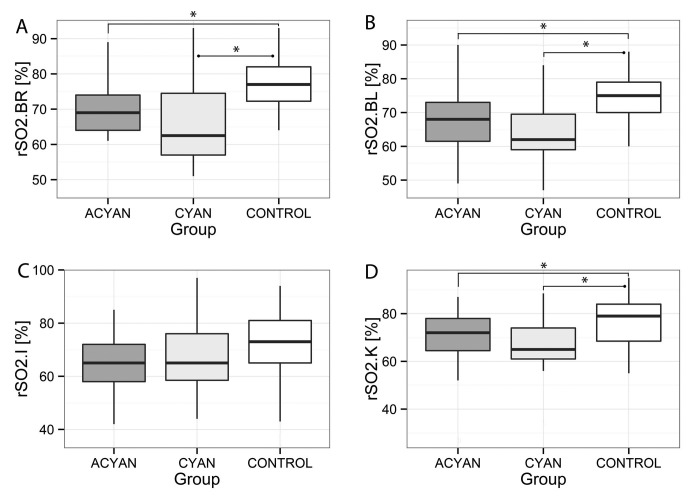
Regional tissue oxygenation (rSO_2_) in the right (**A**) and left brain (**B**), intestine (**C**), and kidney (**D**) in newborns with congenital heart defect (CHD) with cyanosis (CYAN, light gray bars), without cyanosis (ACYAN, dark gray bars), and in the control newborns (CONTROL, white bars). Data are presented as means and standard deviation. *P ≤ 0.01 (Welsh two-sample *t* test).

Newborns with cyanotic CHD had significantly lower median (range) Sao_2_ values than newborns with acyanotic CHD (92% [89%-95%] vs 98% [96%-99%], *P* = 0.006, Table 3); yet, the value was not low enough to affect the average Sao_2_ value of the CHD group as a whole. No other significant differences were found (Table 3). Contrary to what might have been predicted from significantly lower Sao_2_ in cyanotic newborns, the acyanotic and cyanotic newborns with CHD did not differ in rSO_2_ of any tissue (Figure 3).

**Table 3:** Physiological parameters in the newborns with acyanotic and cyanotic congenital heart defect (CHD) (mean ± standard deviation [SD] or median and range)

Parameter	Acyanotic CHD (n = 19)	Cyanotic CHD (n = 16)	Difference (controls-CHD) (95% confidence intervals)	Adjusted *P* (Welsh two-sample *t* test)
	mean ± SD	mean ± SD		
Heart rate (beats/min)	140 ± 17	141 ± 23	0.56 (-13-15)	>0.99
Breathing frequency (breaths/min)	56 ± 13	58 ± 11	2.80 (-5.49-11.08)	>0.99
Arterial blood oxygen saturation (%), median (range)	98 (96-99)	92 (89-95)	-5.88 (-8.88 to -2.87)	0.006
Mean arterial pressure (mmHg)	56 ± 9	59 ± 11	2.60 (-4.66-9.87)	>0.99

## Discussion

Newborns with preoperative CHD had lower rSO_2_ in the brain and kidney than newborns without CHD. Moreover, both groups showed a strong correlation between rSO_2_ of the left and the right hemisphere. Contrary to our hypothesis, no significant differences in rSO_2_ were detected between the cyanotic and acyanotic group.

To the best of our knowledge, this is the first study that assessed and correlated rSO_2_ of different tissues in newborns with various critical cyanotic and acyanotic CHD and compared them to healthy newborns. We hope that these results will contribute to accurate and timely assessment of CHD, which is of crucial clinical importance as patients with CHD exhibit changes in brain structure later in life, resulting in neurologic complications and cognitive and behavioral impairment (1,18,20-22).

Lower brain rSO_2_ in the CHD group than in controls found in our study is in accordance with several other studies (13,20,23). These studies included a very small number of controls or assessed oxygenation in preterm infants, critically ill patients, or children (13,20,23). Contrary to studies (23,24) that mostly included patients with patent ductus arteriosus (PDA), we included patients with different types of CHD. Only Lemmers et al (23) included a comparable number of preterm newborns with PDA and found lower brain rSO_2_ than in healthy newborns; yet, their infants were mechanically ventilated and thus already compromised. We, on the other hand, included newborns who, except cardiac support in terms of diuretics or vasodilators, did not receive any other medication. In fact, the physiological parameters in the CHD group, except for the increased BF, were normal and comparable to controls. Increased BF implies child's distress; in a newborn with CHD, tachypnea is caused by increased pressure in the pulmonary veins resulting from blood flow obstruction to the left ventricle or raised end-diastolic pressure in the left ventricle. It may also be caused by increased fluid volume in the lung bloodstream in large left-to-right shunts (24).

Although the exact limit of the appropriate brain oxygenation as assessed by NIRS is debatable and has not been accurately established (6,7,10,23,25), the significantly lower brain rSO_2_ in the CHD group points to impaired oxygen supply.

Organs other than the brain, which is affected by autoregulation mechanism, might be more seriously affected by inadequate perfusion (26). Several studies tested rSO_2_ of other organs, but none included healthy controls. Amigoni et al (9) monitored the renal, hepatic, and muscular oxygen saturation by NIRS during CHD surgery; Underwood et al (12) proposed the measurement of the kidney and skeletal muscle (deltoid) rSO_2_ as a screen for echocardiographic PDA evaluation; while Owens et al (16) showed a strong correlation between the kidney rSO_2_ and kidney dysfunction in infants with CHD undergoing cardiac surgery. Our study is the first to compare regional oxygenation of tissues other than the brain between newborns with CHD and healthy controls. As expected, the kidney rSO_2_ was lower in newborns with CHD than in controls; also, a significant positive correlation between the brain rSO_2_ and the kidney rSO_2_ was found.

We hypothesized that oxygenation would be lowest in the intestine due to redistribution of blood flow, but did not confirm the hypothesis. The lack of differences in the intestinal rSO_2_ between the CHD group and controls also cannot be explained. The failure to detect lower intestinal rSO_2_ could be attributed to optode placing over a thin neonatal abdominal wall above intestinal loops containing pigments, such as bilirubin, and air; both known to strongly affect the rSO_2_ value (27). Also, the intestine measurements showed greatest variability. More efforts should be directed to the interpretation and objectification of the intestinal rSO_2_ measurements.

Contrary to our hypothesis, despite the trend toward lower oxygenation in the cyanotic subgroup, there were no significant differences in rSO_2_ in any tissue between the cyanotic and acyanotic group. We therefore speculate that newborns with CHD have different hemodynamics than healthy newborns, irrespective of the CHD type. On the other hand, the cyanotic group might have had a low degree of deoxygenated blood shunting, not affecting the oxygenation to a greater extent than in the acyanotic group.

The most important limitations of our study are retrospective design, single center experience, heterogeneous study population, and small sample size. Regarding primary analyses, *post-hoc* power analyses showed that rSO2 comparison between patients with CHD and controls for the kidney and right and left brain hemisphere had power >80%, but our study was underpowered to detect statistical significance for other possibly existing associations. Some other methodological limitations of our study should also be considered. The main problem remains the high spatial, temporal, and inter-subject variability of NIRS (3,6,7,10,12,23,27). Although the use of single point optode remains a concern, the strong correlation of rSO_2_ values between the right and the left hemisphere emphasizes the accuracy of our data. It has also been suggested that the intervention outcome in a specific patient should be assessed, rather than absolute rSO_2_ values (5,10,12,23,28). However, our data confirm the value of absolute values assessment.

In conclusion, we showed that, NIRS was able to detect lower tissue oxygenation and could be regarded as a useful additional non-invasive clinical tool for monitoring and follow-up of newborns at risk for non-optimal brain oxygenation. The most distinctive advantage of NIRS is its non-invasiveness and easy applicability (26). Future long-term follow-up studies using NIRS in newborns are warranted. However, optimal limits of tissue oxygenation in various tissues have to be set. Also, there is a need for devices able to perform an integral determination of rSO_2_ over a larger surface area instead of point probes.
